# Legal, ethical and moral dilemma of human cloning: the conflict between technological progress and human dignity

**DOI:** 10.3389/fmed.2026.1888397

**Published:** 2026-09-03

**Authors:** Jyh-Woei Lin

**Affiliations:** 1 Changzhou Vocational Institute of Mechatronic Technology, Changzhou, Jiangsu, China; 2 Department of Electrical Engineering, Southern Taiwan University of Science and Technology, Tainan, Taiwan

**Keywords:** cloning technologies, four principles of biomedical ethics, human cloning, legal medicine, the future of human nature

## Abstract

Human cloning involves creating a genetically identical copy of a human cell, tissue, or embryo. That is, human reproductive cloning, producing a genetic copy of an existing person, has never been successfully done and remains globally banned or restricted. While there is no scientific evidence of successful human reproductive cloning, biotechnology combines cloning methods with genetic engineering to drive advancements in medical research and disease treatment. With the development of genetic engineering and cloning technologies, human cloning, as a possible future phenomenon, has sparked extensive ethical and moral discussions. In legal medicine, human cloning presents complex challenges intersecting bioethics, criminal law, civil identity, and forensic science. While the technology remains strictly regulated or banned globally, forensic experts and medical jurists analyze cloning through distinct administrative, civil, and criminal frameworks. Human cloning can be ethically evaluated using the foundational four principles of biomedical ethics established by Tom Beauchamp and James Childress including autonomy, beneficence, non-maleficence and justice. These principles provide a widely used framework to analyze the risks, rights, and moral implications of both reproductive and therapeutic cloning. German philosopher Jürgen Habermas strongly opposes human cloning and genetic enhancement because he believes they destroy the foundations of moral equality and personal autonomy. In his seminal work The Future of Human Nature, Jürgen Habermas argues against human cloning and the forensic implications of genetic duplication. He warns that the made status of a cloned individual fundamentally destroys the subject’s capacity to conceive of themselves as an autonomous, undivided author of their own life.

## 1 Introduction

**Figure 1:**
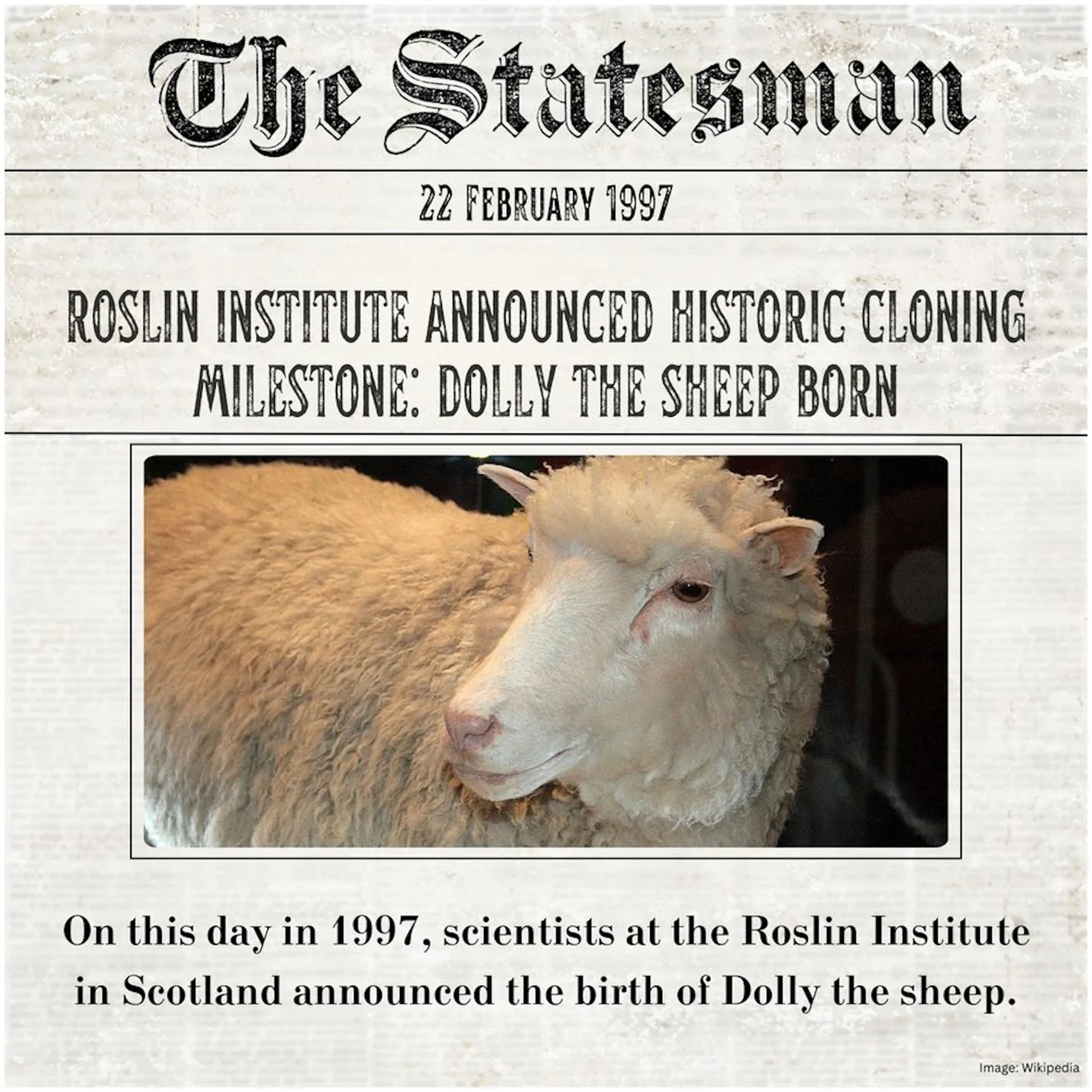
Dolly the sheep in Scotland in 1997.

Over the past few decades, the rapid development of genetic engineering (1–3) and cloning technologies (4–6) has brought the concept of human cloning into the public eye. Genetic engineering (also called genetic modification) is a process that uses laboratory-based technologies to alter the DNA makeup of an organism. That is, Genetic engineering is the deliberate modification of an organism’s genetic material (DNA) using biotechnology. Scientists alter genes to introduce new traits, remove harmful characteristics, or improve biological functions in plants, animals, microorganisms, and humans. Cloning technology refers to techniques used to create genetically identical cells, tissues, or organisms. Legal medicine (7–10), also known as forensic Medicine, is the branch of medicine that applies medical and scientific knowledge to legal matters. It helps courts and law enforcement investigate crimes, determine causes of death, examine injuries, identify victims, and evaluate medical malpractice. Legal medicine includes areas such as forensic pathology, toxicology, and forensic psychiatry, and plays an important role in delivering justice through scientific evidence. In legal medicine, cloning-related technologies are not used to clone humans for legal purposes, but the scientific principles behind cloning and genetic replication play important roles in forensic investigation (11), medical law (12), and criminal justice. In conclusion, cloning technology has significantly influenced legal medicine through advances in forensic genetics, DNA identification, and biomedical research. While it improves the accuracy of criminal investigations and medical analysis, it also creates complex ethical and legal challenges related to privacy, consent, and human rights. As biotechnology develops further, legal systems and medical ethics will continue adapting to regulate its responsible use. The human cloning can be interpreted in multiple ways, biological, technological, philosophical, and fictional. It is not a recognized scientific category, but it raises profound questions about identity, reproduction, and ethics (13). This may involve changing a single base pair (A-T or C-G), deleting a region of DNA or adding a new segment of DNA (14). In addition, cloning technology has significant applications in medicine, particularly in regenerative medicine, disease research, and therapeutic development (15). Regenerative medicine is an interdisciplinary field focused on repairing, replacing, or regenerating damaged cells, tissues, or organs to restore normal function. Unlike conventional treatments that manage symptoms, regenerative medicine aims to address the root cause of disease by rebuilding biological structures. Cloning technology creates genetically identical copies of DNA segments (molecular cloning) (16), cells, or whole organisms. Molecular cloning involves replicating DNA sequences for research, diagnostics, and forensic analysis. Main methods include Somatic Cell Nuclear Transfer (SCNT) (17–19), which produces organisms, and therapeutic cloning to create embryonic stem cells for research. While valuable for medical, agricultural, and research applications, cloning faces ethical and technical challenge. Since the successful cloning of Dolly the sheep in Scotland in 1997 (Figure 1) (20, 21), scientists have been able to use biotechnology to create organisms with identical genes. Human cloning (22–26) is a term that can refer to different concepts depending on the context, ranging from science fiction to emerging technologies and biological processes. The idea of human cloning implies creating exact copies of humans, raising profound ethical and philosophical questions. Humans are cloning in the evolutionary sense, our cells replicate via mitosis, and we reproduce to pass on genetic information. This aligns with Richard Dawkins' concept of the selfish gene, where genes act as cloning driving biological evolution (27–29). That is, the concept is that genes are the fundamental units of natural selection, and organisms are survival machines that pass on these genes, which behave in ways that maximize their replication. The SCNT could theoretically create human clones, but it’s ethically controversial and illegal in many countries. Experimental tech could replicate gestation outside the human body. For biotech replication, advances in synthetic biology aim to engineer cells or tissues (30, 31), but replicating whole human remains far beyond current capabilities. Would a replicated human have the same consciousness, memories, or rights? Could replication be used to create designer humans or perpetuate social hierarchies? Uncontrolled replication could destabilize society or ecosystems. Human cloning not only challenges our fundamental understanding of life but also raise profound questions about individual rights, moral responsibility, and social structures. Moreover, cloning technologies, particularly reproductive cloning, raise profound psychological questions that extend far beyond biology. While cloning is often debated in ethical, legal, and scientific terms, its psychological dimensions are equally significant. These involve identity formation, family dynamics, autonomy, social perception, stigma, moral cognition, and long-term mental health outcomes. Understanding these dimensions is essential if cloning technologies are to be responsibly evaluated or implemented. A central psychological concern surrounding cloning is whether genetic duplication affects personal identity. Psychological theory strongly suggests that identity is not determined solely by DNA (32). Research on monozygotic twins demonstrates that while genetic similarity influences temperament and certain traits, personality, values, and life trajectories diverge significantly due to environmental factors, social experiences, and individual agency. Twin studies strongly support the idea that genetic identity does not strictly dictate psychological identity. The argument from twin studies provides strong evidence that genetic identity does not fully determine psychological identity. By comparing identical twins (who share 100% of their DNA) reared together versus those reared apart, researchers have found that while genetics play a significant role, they do not dictate personality, intelligence, or mental health outcomes in their entirety. Identical twins reared apart often show remarkable similarities, highlighting the profound influence of genetics (33). However, they also display significant differences in their psychological profiles, behaviors, and life choices. This divergence underscores the powerful and nuanced role of non-shared environments, personal experiences, and epigenetics in shaping individual mind (34–39). Correspondingly, the psychology of donor-conceived individuals and adoptees is deeply rooted in the separation from one or both biological parents. While both groups share fundamental themes of identity, genealogy, and ambiguous loss, they experience unique clinical, legal, and developmental dynamics. Usually grow up knowing they are adopted, making the fact a foundational part of their narrative from childhood. Frequently discover the truth late in life through commercial DNA kits. This creates a sudden narrative rupture where their entire biography feels rewritten overnight. Often grapple with the concept of relinquishment or abandonment, the question of why their birth parents could not or chose not to raise them. However, their biological parent chose to provide genetic material, often anonymously and commercially, without the intent to parent. This can lead to feelings of being a product. Searching usually leads to two specific biological parents and perhaps full or half-siblings from those parents (40–45). From the standpoint of developmental psychology, identity formation (Erikson’s stages of psychosocial development) (46) depends on lived experience, social interaction, and personal choices. Erikson’s stages of psychosocial development, proposed by psychoanalyst Erik Erikson, outline eight sequential periods from infancy to late adulthood. Each stage features a core psychosocial crisis that shapes personality and identity; successfully resolving these conflicts fosters healthy development and ego strengths. A cloned individual, despite sharing DNA with another person, would develop a distinct psychological identity shaped by context. Unlike twins, a clone would typically be born after the genetic original has already lived (46). This introduces what some scholars call the predecessor effect (47–49), the psychological burden of being compared to a pre-existing individual. The predecessor effect is a psychological and cognitive phenomenon describing how prior experiences, exposures, or decisions influence subsequent perception, judgment, and behavior. It is widely studied in areas such as decision-making, memory, social comparison, and behavioral economics. While a real cloned human has never lived, applying Erikson’s eight stages highlights unique developmental crises. A clone’s environment dictates their psychological journey, particularly regarding severe expectations from others and their relationship to their genetic original. The challenge is to develop basic trust. A clone raised in a supportive, loving environment will still develop hope. However, the presence of public scrutiny could cause caregivers to over-protect or commodify the child, potentially instilling mistrust in the world. Autonomy requires self-control. If caretakers push a specific blueprint onto the toddler, the child may experience profound doubt and shame when their natural independent actions deviate from the original’s history. A cloned child may feel guilt if their interests or successes do not align perfectly with what others expect from their known genetic template. Clones may face undue pressure to be exceptional at specific tasks (e.g., academics or sports) simply because their donor was. Failing to meet these elevated expectations could lead to intense feelings of inferiority. This is the core psychosocial crisis for a clone. While clones are their own unique people, society might view them as an extension of their genetic donor, hindering their ability to achieve a healthy fidelity to themselves. They will likely struggle to answer “Who am I?” separately from the donor. The predecessor effect is also a concept most commonly discussed in psychology, behavioral science, and learning theory, though its exact meaning can vary slightly depending on context. The predecessor effect describes how earlier experiences, conditions, or events influence later behavior, cognition, or outcomes across psychology, social systems, and research contexts. In psychology, the predecessor effect describes how prior experiences or stimuli influence later perceptions, behaviors, or decisions (50). Potential psychological consequences include, for example, pressure to replicate achievements, feelings of inadequacy, identity foreclosure and existential concerns about authenticity. The psychological challenge is not biological sameness, but social comparison and expectation. It suggests that the core psychological difficulty, for example, in contexts like cloning, twins, or replicated achievement, does not arise from biological similarity itself, but from the social meanings attached to that similarity. Biological similarity, such as identical DNA in twins or clones, does not automatically produce psychological harm. Genetics alone does not determine identity, self-worth, or life trajectory. Individuals with identical genomes can develop very different personalities, interests, and achievements due to environmental, relational, and experiential differences (51). Legal medicine is the application of medical knowledge to legal issues, cases, and proceedings. It bridges healthcare and law, covering areas like medical malpractice, forensic pathology, toxicology, and ethics. This field assists in justice administration, determining causes of death, and investigating criminal or civil cases. For cloning technologies and legal medicine, cloning technologies intersect with legal medicine in complex ways, raising questions about identity, evidence, liability, consent, and the definition of personhood. This relationship spans reproductive cloning, therapeutic cloning, and molecular cloning, each carrying distinct legal medical implications. As stated previously, reproductive cloning involves creating a genetically identical organism through the SCNT, famously demonstrated in Dolly the Sheep. Legal medical relevance includes determination of genetic identity, parentage disputes, inheritance and family law conflicts, birth registration and civil status documentation and human rights and bioethical evaluation. Human rights are the fundamental rights and freedoms inherent to all human beings, regardless of nationality, race, gender, religion, or status. They are grounded in the idea of human dignity, the belief that every person has intrinsic moral worth. A bioethical evaluation is a systematic moral analysis of issues arising from biology, medicine, biotechnology, and life sciences. It examines whether scientific or medical practices respect human dignity, rights, welfare, and justice. Most jurisdictions prohibit human reproductive cloning, including the European Union under the Charter of Fundamental Rights of the European Union (52, 53). Therapeutic cloning creates embryonic stem cells genetically identical to a donor for regenerative medicine. For Legal medical implications, they are, consent for tissue use, embryo status in law, ownership of biological material, malpractice and clinical trial liability and stem-cell–based forensic biomaterials (54). Stem-cell–based forensic biomaterials represent an emerging intersection of tissue engineering, regenerative medicine, and forensic science, utilizing advanced materials to manage, support, and study stem cells for both therapeutic regeneration and potentially forensic identification or analysis. These biomaterials, ranging from natural scaffolds to synthetic polymers, are engineered to mimic the extracellular matrix (ECM) to enhance stem cell survival, differentiation, and integration, thereby improving the quality of tissues. The ECM is a complex network of macromolecules that provides structural and biochemical support to surrounding cells. It is a critical component of all tissues, influencing development, cellular behavior, tissue repair, and disease progression (55). Countries such as the United Kingdom regulate this under the Human Fertilisation and Embryology Act 1990. For relevance to legal medicine, they are DNA amplification in criminal investigations, genetic fingerprinting, paternity testing, biobanking regulations and chain of custody integrity in court proceedings (56, 57).

## 2 Subject

This article mainly aims to explore the legal, the ethical and moral dilemmas involved in the issue of human cloning. As stated previously, a clone, generally speaking, refers to a completely new life form created through gene cloning technology that is genetically identical to an individual. Unlike traditional reproduction methods, cloning technology can bypass natural genetic processes, directly extracting the genome of an individual and generating an identical offspring through asexual reproduction. Asexual reproduction (58–60) is a mode of reproduction in which a single organism produces offspring genetically identical (or nearly identical) to itself, without the involvement of gamete fusion. It is common in microorganisms, plants, fungi, and some animals. Bioethical analysis, which will be discussed later, and legal medicine form an interdisciplinary framework where clinical ethics meet health law. Bioethics defines moral duties and human rights, while legal medicine provides the regulatory mechanisms, liability standards, and statutory enforcement to protect patients, practitioners, and society. Therefore, human cloning intersects legal medicine through bioethics, bodily rights, and regulation. It is universally divided into two categories with vastly different legal, ethical, and medical frameworks: reproductive cloning and therapeutic cloning. Legal medicine serves as the critical bridge between medical science and the justice system. Its primary implication is providing objective, scientifically backed evidence to interpret and resolve legal, civil, and administrative matters. Therefore, these previous statements will be the important issues and discussed in this article.

## 3 Perspective of legal medicine

From the perspective of legal medicine, human cloning sits at a difficult intersection of biomedical science, law, and ethics. Legal medicine focuses on how medical knowledge is applied within legal systems, so when we examine human cloning through this lens, the Main concerns are how biological realities challenge existing legal frameworks governing identity, responsibility, and human rights. Human cloning typically refers to techniques such as the SCNT, the same method used in the creation of Dolly the sheep. In legal medicine, cloning is categorized into reproductive cloning (creating a genetically identical human) and therapeutic cloning (producing embryos for stem cell research) (61). Legal medicine is less concerned with technical feasibility and more with legal consequences of biological duplication. One of the most fundamental legal issues is identity. A clone shares nuclear DNA with the donor but is not the same person biologically and psychologically. Legal systems rely on individual uniqueness for citizenship, criminal liability and inheritance rights. DNA evidence, long considered a gold standard in forensic identification, may lose exclusivity if clones exist. This challenges the reliability of forensic testimony in criminal courts. Legal medicine depends heavily on biological markers including DNA profiling, fingerprints, dental records and cloning introduces genetic duplication, which complicates forensic conclusions (62). Therefore, legal medicine would need to evolve beyond DNA determinism, incorporating multi-layered identification techniques (63). Therefore, human cloning is one of the most controversial developments in legal medicine. It raises profound questions concerning identity, personhood, human dignity, reproductive rights, medical responsibility, and legal regulation. From the perspective of legal medicine, human cloning is not merely a scientific issue but a multidisciplinary challenge involving medicine, law, ethics, forensic science, and public policy. Legal medicine examines how cloning affects legal rights and obligations, forensic identification, medical liability, inheritance law, family relationships, and criminal justice systems. This paper explores human cloning through the lens of legal medicine, analyzing its scientific foundations, ethical concerns, legal implications, forensic challenges, and regulatory frameworks. The study argues that while therapeutic cloning may offer significant medical benefits, reproductive human cloning presents substantial legal and ethical risks that current legal systems are inadequately prepared to address. Consequently, legal medicine supports a precautionary regulatory framework that protects human dignity while permitting carefully regulated scientific research.

## 4 Bioethical analysis

Corresponding to the perspective of legal medicine, a bioethical analysis for the topic in the article is also important because the both are associated and inseparable (64–66). That is, the both are deeply interconnected fields that evaluate the moral, human rights, and legal dimensions of healthcare. While bioethics provides the philosophical framework for determining what ought to be done, legal medicine translates these moral standards into enforceable laws, malpractice regulations, and forensic procedures to protect patient rights and societal welfare (66). A bioethical analysis is a structured way of examining moral questions in medicine, biotechnology, and life sciences. It helps clarify what should be done when scientific possibilities raise difficult human, legal, and societal issues. Bioethical analysis evaluates actions, policies, or technologies by applying ethical principles, philosophical reasoning, and sometimes legal standards. It is commonly used in areas like gene editing (e.g., the Clustered Regularly Interspaced Short Palindromic Repeats, CRISPR) (67, 68), end-of-life care (euthanasia, physician-assisted suicide), human cloning, organ transplantation and AI in healthcare. Most modern bioethical analysis is grounded in four widely accepted principles by Tom Beauchamp and James Childress (69): (1) autonomy: respecting a person’s right to make their own decisions and requires informed consent and freedom from coercion, (2) beneficence: acting in the patient’s best interest and promoting wellbeing and positive outcomes, (3) non-maleficence: do no harm and avoid unnecessary risk or injury, (4) justice: fair distribution of resources, risks, and benefits and addresses inequality and access to care Ethically permissible with strict regulation for therapeutic purposes, but controversial for enhancement. However, there are some challenges in bioethical analysis, e.g., cultural and religious differences, rapid technological advancement outpacing regulation and uncertainty in long-term consequences. The bioethical analysis of human cloning sits at the intersection of science, law, and moral philosophy. It draws heavily on foundational principles in bioethics and is shaped by debates in genetic engineering and reproductive technology. The discussion typically distinguishes between reproductive cloning (creating a genetically identical human being) and therapeutic cloning (creating embryos for research or medical use). These should be also important principles based on the legal medicine (70–73). These four principles of biomedical ethics provide a useful framework for evaluating the ethical implications of human cloning. These principles are autonomy, beneficence, non-maleficence, and justice. Autonomy refers to respecting individuals’ rights to make informed decisions about their own lives and bodies. In the context of human cloning, prospective parents may claim a right to reproductive autonomy, arguing that they should be free to use cloning technologies to have genetically related children. However, concerns arise regarding the autonomy of the cloned individual. A clone cannot consent to being created through cloning and may face expectations based on the life of the genetic donor. Knowledge of one’s genetic origin may influence personal identity formation and self-determination. Human cloning could compromise the clone’s ability to develop an independent identity and exercise full autonomy. Beneficence requires actions that promote the welfare and wellbeing of others. Potential benefits of cloning including helping infertile individuals have genetically related children, allowing reproduction when one parent cannot produce viable gametes and advancing biomedical research that may lead to new therapies and treatments. However, these benefits must be weighed against potential harms and uncertainties. The anticipated benefits of cloning remain largely speculative, while the risks to cloned individuals are substantial and not fully understood. Non-maleficence means do no harm. This principle is particularly significant in discussions of human cloning. Research involving animal cloning has demonstrated, i.e., high rates of miscarriage and embryonic loss, developmental abnormalities, increased risk of premature aging and disease and significant suffering among cloned animals. If similar risks were present in humans, cloning could expose both the clone and the gestational mother to unacceptable harm. Given current scientific limitations, reproductive human cloning would likely violate the principle of non-maleficence by creating foreseeable risks of physical and psychological harm. Justice concerns fairness, equality, and the equitable distribution of benefits and burdens. Human cloning raises several justice-related issues. Access to cloning technology would likely be limited to wealthy individuals, potentially increasing social inequality. Clones could face discrimination, stigmatization, or unequal treatment. The commercialization of cloning may encourage the treatment of human life as a product. Resources devoted to cloning might divert attention from more pressing public health needs. Human cloning may create new forms of inequality and social injustice while concentrating benefits among a privileged minority.

## 5 Current gene and cell therapy research

Gene therapy research aims to treat or cure diseases by replacing, inactivating, or introducing genes into a patient’s cells (74). Driven by clustered regularly interspaced. Cell therapy involves transferring living, modified, or engineered cells into a patient to treat diseases, combining immunology and regenerative medicine. Many treatments are transitioning from laboratories into clinical trials Gene and cell therapy research is one of the fastest-evolving areas in modern biomedicine, focused on treating, or even curing, diseases by modifying genes or using living cells as therapeutic agents (67, 75). Scientific and transnational integration in current gene and cell therapy research is not just a trend. It is the backbone of how these therapies are advancing from experimental science into real-world medicine. The field depends heavily on global collaboration, shared standards, and cross-border regulatory alignment because of its complexity, cost, and ethical implications. Modern gene and cell therapy sits at the intersection of several scientific domains. Enables precise genome editing and is widely used across countries in both basic research and clinical trials. Global datasets (genomics, proteomics) are shared across platforms. AI-driven drug discovery and patient stratification enhance therapy precision. Initiatives like the Human Genome Project laid the foundation for global genomic collaboration.

## 6 Ethical principles of cloning technology

Based on the aforementioned ethical issues, ethical principles concerning cloning technology have been proposed in this study. These principles especially emphasize the following aspects: (1) Respect for individual dignity: Respect for individual dignity is a foundational principle that every human being possesses inherent worth and should be treated with consideration, fairness, and moral regard, simply by virtue of being human. Every clone should be regarded as an individual with intrinsic value, rather than simply a genetic copy. (2) Protect individual freedom and right to choose: Protecting individual freedom and the right to choose is a foundational principle of democratic societies, human rights law, and modern ethics. It affirms that individuals should be free to make decisions about their own lives without unjust interference, as long as they do not harm others. Human cloning should have the same right to choose as other humans, whether in family, education, or career. (3) Preventing the misuse of technology: Preventing the misuse of technology is a central concern in modern ethics, law, and public policy. As technologies such as artificial intelligence (AI), biotechnology, surveillance systems, digital platforms grow more powerful and the potential for harm increases alongside their benefits (76). Cloning technology should be strictly regulated to prevent it from being used for military and political purposes, and then to ensure that clones are not used as tools or items. Significant differences exist in the legislation regarding human cloning among countries. Some countries completely prohibit cloning technology, while others allow research under certain specific circumstances (77). However, global regulation on this issue remains inconsistent, necessitating the development of more specific international norms and legal frameworks to safeguard fundamental human ethical standards. Fundamental human ethical standards are the core principles that guide moral behavior across cultures and societies (78).

## 7 Legal, ethical and moral dilemma of human cloning

### 7.1 Legal landscape and dilemma of human cloning

International consensus currently rejects human reproductive cloning. The United Nations Declaration on Human Cloning (2005) (79) calls for prohibition, though it passed through a divided vote and lacks binding force. The declaration was adopted on March 8, 2005, by the UN General Assembly. It addresses the ethical, legal, and social concerns surrounding human cloning technologies. Unlike a treaty, it is not legally binding, but it reflects global ethical guidance and political consensus (though not unanimous). The declaration calls on states to prohibit. All forms of human cloning insofar as they are incompatible with human dignity and the protection of human life. This wording is deliberately flexible, allowing different interpretations. Safeguard human life in the application of life sciences. Prevent exploitation of human beings in biotechnology. There is broad international consensus against reproductive cloning. This is seen as ethically problematic due to risks, identity concerns, and dignity issues. Some countries support it for medical advancement. Others oppose it due to embryo destruction concerns. The declaration avoids a strict universal ban, reflecting this divide. Countries are not legally required to follow it and allow different national interpretations. Reflects political compromise rather than strict ethical agreement (80–82). The Council of Europe's Oviedo Convention explicitly bans reproductive cloning (83), while permitting some therapeutic research under strict controls. National approaches vary dramatically. Germany's Embryo Protection Act was enacted on December 13, 1990, and entered into force on January 1, 1991 (84). It represents one of the strictest regulatory frameworks for reproductive medicine in Europe, establishing criminal penalties for various prohibited practices. Germany’s Embryo Protection Act criminalizes any attempt to create genetically identical humans, with penalties up to 5 years imprisonment. The United Kingdom permits therapeutic cloning under regulatory oversight while banning reproduction (85). Replication tourism (86), traveling to another country to access assisted reproductive technologies such as surrogacy potentially future cloning-related procedures, comes with a complex mix of medical, legal, ethical, and psychosocial risks. Regulations differ widely across countries. Some clinics may not meet the safety standards enforced in places like the European Union (EU) or Singapore. Donors or surrogates may not be thoroughly screened for genetic or infectious diseases (87). Patients may return home before complications arise, leaving local doctors without full medical records. In less-regulated settings, unproven or ethically controversial techniques may be offered (88). Replication technology generates fundamental tensions within human rights law. Proponents might argue that access to replication falls within protected reproductive autonomy, the right to found a family recognized in international instruments (52, 89–91). Courts have broadly interpreted reproductive rights regarding contraception and abortion; cloning represents a logical, if extreme, extension (92). However, replication directly conflicts with the right to genetic identity (93, 94) protected in the Convention on the Rights of the Child and various bioethics frameworks (95, 96). Unlike natural reproduction’s genetic lottery, deliberate copying eliminates uniqueness as a matter of design. How do legal systems reconcile competing rights when the harm is inherent to the technology itself? Human dignity is a fundamental concept in ethics, philosophy, law, and human rights that refers to the inherent and inalienable worth of every human being (97). It is the idea that all people possess a special value simply by virtue of being human, regardless of their social status, abilities, achievements, or any other external factors. The human dignity argument presents similar paradoxes. German constitutional law revolves primarily around the Grundgesetz (Basic Law), which is the constitution of the Federal Republic of Germany. It was adopted in 1949 as a provisional constitution for West Germany and became the constitution of unified Germany in 1990 (98). The Basic Law functions as the supreme law of the land. All federal and state laws must comply with it. German constitutional law is centered on the Basic Law (Grundgesetz), which has served as Germany's constitution since 1949. German constitutional law has proven remarkably stable and influential, serving as a model for other post-authoritarian constitutions worldwide. European bioethics frameworks operate through a multi-layered system involving both the Council of Europe and the EU, though with distinct competencies and approaches. The Council of Europe's Oviedo Convention provides broad human rights principles for biomedicine across its member states (including non-EU countries), while the EU focuses on specific regulatory harmonization where treaty competence exists, particularly regarding market authorization, data protection, and research funding ethics requirements (99–101). Therefore, German constitutional law and European bioethics frameworks prohibit treating humans as mere means. Replication arguably instrumentalizes the copied individual. Perhaps no legal question proves more destabilizing than determining the status of replicated humans. Such law assumes genetic uniqueness as foundational to identity verification. DNA evidence, paternity testing, and forensic identification all rely on this distinctiveness. In addition, the Future of Human Nature, German philosopher Jürgen Habermas addresses the paradox of liberal eugenics, the idea that genetic selection and enhancement can be morally permissible if driven by the autonomous, individual choices of parents, rather than an authoritarian state (102). Proponents of liberal eugenics argue that parents should be free to select the genetic traits of their children to give them a better chance at life. Habermas argues that this inherently undermines the foundational liberal principle of individual autonomy. Unlike environmental shaping (like putting a child in piano lessons), genetic design is an irrevocable, asymmetrical intervention where one generation permanently determines the baseline physical or psychological traits of the next. For a person to be morally autonomous, they must be able to view themselves as the sole author of their life choices. If a person discovers that fundamental aspects of their biological makeup were pre-programmed by the deliberate choices of another person, they can no longer fully regard their body and life plan as entirely their own. By shifting reproduction from the realm of contingency to the realm of production, Habermas warns that we risk blurring the line between human beings and artifacts. Once human traits become subject to technical manipulation and parental preference, children risk being viewed as extensions of parental will or consumer products, rather than equal beings born without prior conditions. This commodification threatens the ethical self-understanding of the species potentially altering the egalitarian relationships between free and equal individuals upon which democratic societies rely (103–105). In his book The Future of Human Nature, German philosopher Jürgen Habermas examines the ethical implications of human genetic engineering and cloning. Although Habermas focuses primarily on genetic enhancement, his arguments provide a significant critique of human cloning because both practices involve intentional intervention in the biological makeup of future persons. Habermas argues that every person should enter the world as a morally autonomous individual who can regard themselves as the author of their own life. Human cloning challenges this principle because a clone is deliberately created from the genetic blueprint of an existing person. According to Habermas, this creates an asymmetrical relationship between the creator and the cloned individual. The clone may perceive that important aspects of their identity were intentionally predetermined by others. This could undermine the clone’s sense of freedom and equality, which are essential foundations of modern democratic societies. Habermas draws upon the moral philosophy of Immanuel Kant, particularly the idea that human beings must always be treated as ends in themselves rather than merely as means to an end. Human cloning raises concerns that individuals might be created for specific purposes, such as replacing a deceased child, providing compatible organs or tissues, fulfilling parental expectations and replicating desirable traits. In such cases, the cloned person risks being valued primarily for the purpose they serve rather than for their inherent dignity as a unique individual. Habermas emphasizes that personal identity develops through an open and unpredictable interaction between genetics, environment, and individual choices. Human cloning may alter this process because the clone’s genetic identity is intentionally selected. Even though clones would not be exact copies psychologically or socially, knowledge of sharing the genome of another person could create pressures and expectations regarding such as personality traits, talents and abilities, career choices and social roles. Habermas worries that such expectations could interfere with the clone’s ability to develop an independent self-understanding. A main element of Habermas’s argument is that democratic societies depend on the assumption that citizens relate to one another as free and equal moral agents. Human cloning introduces a new form of human design in which one person deliberately determines the biological characteristics of another. Habermas argues that this may weaken the moral symmetry that underpins human rights and democratic equality. The clone may occupy a position in which aspects of their biological identity were chosen by others, creating a relationship of dependency not present in ordinary reproduction. Genetic interventions for therapeutic purposes, intended to prevent serious disease. Genetic interventions for enhancement or design, intended to shape desired traits. While he cautiously accepts some therapeutic interventions that can be presumed to serve the future child’s interests, he strongly criticizes interventions that aim to design future persons according to parental or social preferences. Human cloning generally falls into this latter category because it involves deliberate selection and reproduction of a particular genome. Some scholars argue that Habermas overestimates the influence of genetics on identity. Critics note that identical twins share the same genome yet develop distinct personalities, environmental and social factors strongly influence personal development and a clone would still possess free will and moral agency. Others contend that cloning does not necessarily undermine autonomy if the cloned individual is treated with dignity and granted the same rights as any other person. From a legal medicine perspective, Habermas’s analysis supports strict regulation of reproductive cloning because of concerns regarding such as human dignity, personal autonomy, informed consent of future persons, psychological welfare of cloned individuals and protection against genetic discrimination and exploitation. His work has influenced broader international debates reflected in documents such as the Universal Declaration on the Human Genome and Human Rights, the United Nations Declaration on Human Cloning, and the Oviedo Convention, all of which emphasize the protection of human dignity in the face of emerging biotechnologies.

### 7.2 Ethical and moral dilemma of human cloning

Kant's ethical and moral theories, known as deontology or the categorical imperative (106–109), is one of the most influential moral philosophies in Western thought. According to Kant's ethical theory, every individual should be considered a being with intrinsic dignity and should be respected by others. One of the core ethical challenges of the cloning issue is whether clones can obtain the same respect and autonomy as other humans. Kant’s moral theory is a deontological, duty-based system emphasizing that moral worth depends on motivations (a good will) rather than consequences. In Kant’s moral theory, good will refers to the only thing that is unconditionally good (110–112). According to Immanuel Kant, a good will is good not because of what it effects or accomplishes, but by virtue of its willing alone, meaning the intention to act from duty, regardless of outcomes. Good Will includes friendly, helpful, or cooperative feelings or attitude, benevolent intention or disposition toward others and the positive reputation or rapport a person or organization enjoys. It centers on the categorical imperative, an unconditional, rational law requiring that actions be universalizable and that humans are always treated as ends in themselves, never merely as means (113, 114). Since clones are genetically identical to the original individuals, whether they should be considered a self or simply a copy warrants further exploration? Can clones be considered independent individuals with autonomy, capable of freely choosing their lives? If not, does this violate the fundamental requirement of individual dignity in modern ethics? Genetically, a clone is a complete copy of the original individual, which may prevent them from being emotionally and psychologically independent of their original model individual. This can not only affect the clone’s own identity but also have a profound impact on their relationships with family members. Whether clones can truly integrate into traditional family structures and receive equal love and support remains a question worth considering. Can clones possess the same emotional and social status as non-clones? Can they overcome potential rejection and misunderstanding from family and society? If cloning technology is used to create humans with the same genes as a specific individual, purposeful cloning may occur. For example, some governments or institutions may create clones for political, economic, or military purposes. This can refer to several different topics including human cloning and animal cloning (115). Clone armies in fiction (e.g., Star Wars clone troopers). Digital clones or AI replicas (116, 117) (e.g., creating digital replicas of people for influence or propaganda). Biological weapons or engineered organisms (e.g., using cloning technology for military applications). Cloning technology for military applications is a controversial and highly speculative topic that intersects biotechnology, ethics, international law, and security studies (4, 118). While popular culture often portrays cloned soldiers or enhanced human replicas, the scientific and legal realities are far more complex. Such situations not only raise ethical issues but also lead to the objectification of individuals. Objectification of individuals refers to the treatment of a person as a thing, valued primarily for their utility, appearance, function, or productivity rather than for their full humanity, autonomy, and dignity. It occurs in social, cultural, economic, political, and technological contexts. When clones exist solely for some external purpose, it jeopardizes their fundamental dignity and human rights as human beings. Will clones be objectified or exploited? Will the value of their lives be severely diminished? Reproductive cloning is universally condemned by global medical and ethical bodies due to extreme health risks and violations of human dignity. The process carries an exceptionally high failure rate, resulting in miscarriages, stillbirths, and severe genetic abnormalities in animals, posing unacceptable physical and psychosocial dangers to any resulting human. The global legal landscape governing therapeutic cloning is highly fragmented (119). While human reproductive cloning is broadly prohibited by international consensus, therapeutic cloning (using the SCNT to harvest stem cells for medical research) faces a patchwork of regulations ranging from outright bans to permissive, heavily regulated frameworks. The psychological burden on cloned individuals would likely revolve around threats to personal identity, a lack of genetic uniqueness, and relentless public scrutiny. Because they share the exact genome of a predecessor, clones would be at risk for a predetermined life script and extreme psychological expectations. Therefore, human cloning presents profound challenges for forensic science because many forensic identification techniques rely on the assumption that each individual possesses a unique genetic profile. If human cloning were to become feasible, genetically identical individuals could complicate criminal investigations, paternity disputes, victim identification, and the administration of justice. From the perspective of legal medicine and forensic genetics, human cloning raises important questions regarding the reliability of DNA evidence and the legal systems that depend upon it. Human cloning, particularly reproductive cloning through techniques such as the SCNT, would produce an individual whose nuclear DNA is nearly identical to that of the donor. This process was demonstrated in the cloning of Dolly the Sheep in 1996. Although no verified case of human reproductive cloning exists, advances in biotechnology continue to stimulate ethical and legal debate. A cloned individual would share essentially the same nuclear genome as the donor, much like naturally occurring identical twins. However, environmental influences, epigenetic modifications, and life experiences would produce differences over time. Despite these differences, conventional forensic DNA profiling might not be able to distinguish between a clone and the original genetic donor. Modern forensic science relies heavily on DNA profiling to identify suspects and exclude innocent individuals. DNA evidence is often considered one of the most reliable forms of forensic proof because the probability of unrelated individuals sharing the same DNA profile is extremely low. Human cloning would challenge this assumption. If a crime scene sample matched a cloned individual’s DNA profile, investigators might be unable to determine whether the biological material originated from the clone or the genetic donor. This could create reasonable doubt in criminal proceedings and weaken the evidentiary value of DNA matches. Although clones share the same genome, forensic scientists could potentially differentiate individuals through epigenetic markers such as DNA methylation patterns, differences in mitochondrial DNA if donor eggs originate from different individuals, fingerprints, which are influenced by prenatal environmental factors and are unique even among identical twins, biometric characteristics such as retinal patterns, facial morphology, and voice patterns and environmental and lifestyle-related biological markers. Future forensic techniques may therefore shift from purely genetic identification toward a combination of genetic and phenotypic evidence. A cloned individual would be a distinct legal person despite possessing the same genetic makeup as another individual. Consequently, criminal responsibility would remain individual rather than genetic. However, genetic duplication could complicate establishing the identity of perpetrators, determining the source of biological evidence, investigating cases involving fraud or identity theft and proving presence at a crime scene. Courts would need to recognize that DNA evidence alone may be insufficient in cases involving clones and would likely require corroborating evidence such as surveillance footage, witness testimony, digital records, and biometric data. Forensic genetics plays a crucial role in identifying victims of mass disasters, armed conflicts, and missing-person cases. Human cloning could complicate these efforts because DNA samples collected from remains might correspond to multiple genetically identical individuals. Forensic investigators would need to rely more heavily on dental records, medical implants, skeletal characteristics, fingerprints and personal belongings and contextual evidence. This would reinforce the multidisciplinary nature of disaster victim identification within legal medicine. Human cloning would also affect kinship analysis. Traditional genetic relationships become more complex because a clone is genetically similar to the donor while belonging to a different generation. Questions could arise regarding legal parenthood, inheritance rights, custody disputes and family relationship determinations. Forensic geneticists might encounter difficulties distinguishing between a donor and a clone in paternity or identity testing, requiring more advanced molecular analyses and legal definitions of family relationships. Many countries maintain forensic DNA databases to assist law enforcement. Human cloning raises concerns about genetic privacy because a clone’s DNA profile could reveal information about the donor and vice versa. Potential issues include misidentification within criminal databases, unauthorized use of genetic information, genetic surveillance concerns and challenges in maintaining accurate identity records. Regulators would need to establish safeguards ensuring that genetic similarity does not result in mistaken accusations or violations of individual rights. The forensic implications of cloning strengthen ethical arguments against human reproductive cloning. International instruments such as the Council of Europe’s Oviedo Convention and the United Nations Declaration on Human Cloning emphasize the protection of human dignity and caution against practices that may undermine individuality and human rights. Traditional forensic science has long relied on the uniqueness of genetic information as a means of personal identification. Human cloning would require forensic systems to adopt broader approaches that integrate genetics, epigenetics, biometrics, and contextual evidence. Summarily, the analysis of this article demonstrates that reproductive human cloning poses substantial risks to human dignity, personal autonomy, psychosocial identity development, and legal accountability. Consequently, a viable regulatory framework should prohibit reproductive cloning, permit therapeutic cloning only under stringent ethical oversight, establish international governance mechanisms, protect the rights of any cloned individuals, and develop new forensic identification standards capable of addressing genetic duplication. Such a framework balances scientific innovation with the protection of fundamental human rights. A viable framework requires can be a multi-tiered model combining (1) dignity-based substantive limits grounded in Kantian ethics, the Oviedo Convention and the UN Declaration (2) deliberative democratic legitimacy as articulated by Habermas, (3) psychosocial safeguards informed by Eriksonian identity theory, and (4) enforceable legal-medical traceability mechanisms derived from forensic genetics. Together, these elements suggest a hybrid regulatory architecture: strict prohibition of reproductive cloning and tightly controlled research permissions subject to transnational ethical deliberation, as well as robust identity protection regimes for any potential future applications. This four-part model is a smart way to make rules for human cloning. It uses philosophy, psychology, law, and science to protect people while letting safe research happen. They are the new finding of this article.

In addition, corresponding to human cloning technologies, we consider the ethical perspectives of the German philosopher Hans Jonas, who extensively examined the moral implications of advanced technologies, human cloning raises profound questions concerning human dignity, identity, autonomy, and the limits of technological intervention. In works such as The Imperative of Responsibility and Technology, Medicine and Ethics, Jonas argues that technological capabilities must be accompanied by a heightened sense of ethical responsibility, particularly when innovations possess the potential to fundamentally alter the conditions of human existence. Applying Jonas’s framework to human cloning highlights the need to consider not only scientific feasibility but also the long-term consequences for individuals and society (120–124). Although some countries, including Germany, have developed relatively comprehensive legal restrictions on cloning-related research and practices, the broader European context presents significant regulatory difficulties. The increasing mobility of people, scientific collaboration across borders, and the relatively unrestricted movement within the EU create potential gaps in enforcement and regulatory consistency. Consequently, isolated national regulations may be inadequate to address the transnational nature of human cloning technologies. A more effective governance framework requires coordinated legal and political mechanisms at the European and international levels, balancing scientific advancement with the protection of human rights, human dignity, and ethical responsibility (125–128). The transhumanist movement is worth discussing although it is not corresponding to human cloning technologies (129, 130). The transhumanist movement argues that humanity should use advanced science and technology to transcend biological limitations. Among its most influential advocates, Nick Bostrom and Julian Savulescu have proposed that genetic engineering, reproductive technologies, and other forms of human enhancement could significantly improve human wellbeing. While their positions differ in important respects, the both suggest that biotechnology may 1 day reduce physical, cognitive, and even certain moral limitations traditionally regarded as part of human nature (88, 131–133).

## 8 Conclusion

The debate over human cloning highlights a deep friction between scientific advancement and ethical boundaries. Framing this complex issue around types of cloning, legal status, and medico-legal implications provides a clear way to understand why it remains one of the most heavily scrutinized topics in modern science and legal medicine. To understand its medico-legal implications, the issue must be broken down by the types of cloning, their global legal status, and the resulting forensic and ethical challenge. Opponents argue cloning treats individuals as a means to an end and infringes on a clone’s right to an open future and a unique identity. Proponents, however, advocate for the utilitarian medical benefits that therapeutic cloning could yield for treating incurable degenerative diseases. The issue of cloning is a microcosm of the conflict between modern technology and ethics. While scientific progress offers us many possibilities, we must be cautious in protecting human dignity and ethical principles while promoting technological development. In the future, the regulation of cloning technology must place greater emphasis on ethics, legality, and humanity to ensure that technological progress truly benefits all humankind. Finally, in conclusion, the analysis of this article demonstrates that human cloning cannot be adequately governed through either prohibitionist declarations or fragmented biomedical regulation. A viable framework requires a model combining (1) dignity-based substantive limits grounded in Kantian ethics, the Oviedo Convention and the UN Declaration (2) deliberative democratic legitimacy as articulated by Habermas, (3) psychosocial safeguards informed by Eriksonian identity theory, and (4) enforceable legal-medical traceability mechanisms derived from forensic genetics. Together, these elements suggest a hybrid regulatory architecture: strict prohibition of reproductive cloning and tightly controlled research permissions subject to transnational ethical deliberation, as well as robust identity protection regimes for any potential future applications.

## In memoriam

In memory of my mother who was dead on 09 October 2016.
